# Variable clinical phenotypes of alpha‐methylacyl‐CoA racemase deficiency: Report of four cases and review of the literature

**DOI:** 10.1002/jmd2.12437

**Published:** 2024-07-11

**Authors:** Arzu Selamioğlu, Mehmet Cihan Balcı, Meryem Karaca, Youssef Khalil, Rohit Hirachan, Hacer Durmuş Tekçe, Yeşim Gülşen Parman, Asuman Gedikbaşı, Mübeccel Demirkol, Peter Clayton, Gülden Gökçay

**Affiliations:** ^1^ Bağcılar Training and Research Hospital Division of Pediatric Metabolic Diseases Istanbul Turkey; ^2^ Department of Rare Diseases, Institute of Graduate Studies in Health Sciences Istanbul University Istanbul Turkey; ^3^ Division of Pediatric Nutrition and Metabolism Istanbul University Faculty of Medicine Istanbul Turkey; ^4^ Inborn Errors of Metabolism, Genetics and Genomic Medicine UCL Great Ormond Street Institute of Child Health London UK; ^5^ Department of Neurology Istanbul University Faculty of Medicine Istanbul Turkey; ^6^ Division of Medical Genetics, Department of Pediatric Basic Sciences, Institute of Child Health Istanbul University Istanbul Turkey

**Keywords:** alpha‐methylacyl‐CoA racemase deficiency, *AMACR* gene, hypocholesterolemia, peroxisomal disorders, rhabdomyolysis, variable phenotype

## Abstract

Alpha‐methylacyl‐CoA‐racemase (AMACR) deficiency (MIM#604489) is a peroxisomal disorder resulting in the accumulation of pristanic acid, dihydroxycholestanoic acid (DHCA), and trihydroxycholestanoic acid (THCA), with variable clinical features and age of onset from infancy to late adulthood. The purpose of this report is to define clinical variations and follow‐up data in AMACR deficiency emphasizing treatment with a review of cases reported in the literature. Here, four patients, from two families, diagnosed with AMACR deficiency and showing phenotypic heterogeneity are presented. A 10‐month‐old‐female presented with coagulopathy, hepatic dysfunction, and elevated pristanic acid, DHCA, and THCA levels. Genetic testing confirmed a homozygous variant c.596G>A in the *AMACR* gene. Her brother who had macrovesicular hepatosteatosis and elevated pristanic acid levels was diagnosed with family screening. The third patient presented with rhabdomyolysis following a strenuous exercise without any other complaint. Homozygous novel c.1006G>A variant was found on the *AMACR* gene. His asymptomatic sister carrying the same variant also had elevated pristanic acid levels. They had normal neuropsychologic evaluation. Dietary treatment with low phytanic and pristanic acid content was recommended to the patients but all showed poor compliance. The sibling pairs were followed for periods of 11 and 7 years, respectively. AMACR deficiency is usually described as an adult‐onset disorder with neuropsychological problems. The characterization of natural history and new clinical phenotypes may support earlier diagnosis and treatment.

**Synopsis**

This is the first report of AMACR deficiency where there is a phenotypic heterogeneity in the same family.

## 1 INTRODUCTION

Alpha‐methylacyl‐CoA‐racemase (AMACR) deficiency (MIM#604489) is a rare autosomal recessive peroxisomal disorder. AMACR facilitates the interconversion of (*R*)‐ and (*S*)‐stereoisomers of α‐methyl‐branched‐chain fatty acyl‐CoA esters, including pristanoyl‐CoA, the CoA esters of dihydroxycholestanoic acid (DHCA), trihydroxycholestanoic acid (THCA), which are the only stereoisomers that can be degraded via peroxisomal β‐oxidation. AMACR deficiency results in the accumulation of pristanic acid, DHCA, and THCA. It is caused by biallelic variants in the *AMACR* gene.^1^ The clinical characteristics of AMACR deficiency vary with age. It may present as a late‐onset form with sensorimotor neuropathy, pigmentary retinopathy, seizures, tremor, cerebellar ataxia, cataract, type 2 diabetes, and relapsing encephalopathy in adults. An early presentation at infancy is associated with abnormal bile acid synthesis, coagulopathy, and neonatal cholestasis.^1^, ^2^, ^3^ AMACR deficiency was first described in 2000, and in the literature, less than 20 genetically confirmed cases were reported so far.^1^ We report the clinical and metabolic phenotypes and follow‐up features of four patients diagnosed with AMACR deficiency confirmed by molecular genetic analysis, as they expand the clinical spectrum of this ultra‐rare disease and indicate phenotypic heterogeneity in the same family.

## 2 CASE REPORTS

### 2.1 Patient A1

The first patient was born to consanguineous Turkish parents after an uneventful term pregnancy. Birth history was unremarkable with a birth weight of 3150 g. Growth and development was normal until she presented due to abdominal distension and tendency to ecchymosis at the age of 10 months. Physical examination revealed minimal hepatosplenomegaly. Laboratory workup revealed liver dysfunction with a mild elevation of plasma aspartate transaminase (AST) (88 U/L, normal range (nr): 5–40), alanine transaminase (ALT) (70 U/L, nr: 10–40), creatine kinase (CK) (344 U/L, nr: 30–200), prolonged prothrombin time (18.7 s, nr: 11–16.8), and international normalized ratio (INR) (1.48, nr: 0.8–1.25). Plasma cholesterol levels were low (total cholesterol: 96 mg/dL [nr: 130–200], low‐density lipoprotein cholesterol: 57 mg/dL [nr: 100–130], high‐density lipoprotein cholesterol: 27 mg/dL [nr > 40], triglycerides: 57 mg/dL [nr: 0–150]; Table 1). Metabolic tests including a dried blood spot analysis of amino acids and acylcarnitines by tandem mass spectrometry (MS/MS), quantitative plasma amino acids, and urinary organic acids by gas chromatography–mass spectrometry (GC–MS) were normal except for pathologic plasma pristanic acid. Bile acid intermediates in dried blood spot showed normal primary and secondary C24 bile acids and their conjugates but significantly elevated C27 bile acids (DHCA = 10.96 μmol/L, nr <0.04; THCA = 0.59 μmol/L, nr <0.04) raising the suspicion of peroxisomal dysfunction. The analysis of the stereoisomers of THCA disclosed an almost complete absence of the *S*‐isomer of both taurine‐THCA and free THCA (<1%) and a clear accumulation of the *R*‐isomers (>99%) which was highly suggestive for AMACR deficiency. Very long chain fatty acids (VLCFA) analysis in plasma showed normal to low levels of C26, C24, and C22 fatty acids while plasma pristanic acid was 12.8 μmol/L (nr: 0–1.5), indicating a significant elevation (Table 1). Abdominal ultrasonography (USG) showed increased echogenicity of liver. Liver biopsy showed hepatocellular degeneration and regeneration without fibrosis. Echocardiography (ECHO), electromyography (EMG), and ophthalmological evaluations were normal. Cranial magnetic resonance imaging (MRI) revealed nonspecific millimetric signal density increase in right frontoparietal deep white matter. Genetic analyses revealed homozygosity for variant c.596G>A (p.Cys20Tyr) on the *AMACR* gene. The cysteine at position 20 is invariably conserved among AMACR proteins from multiple species. The parents were carriers of this variant. Fat‐soluble vitamin supplements and a diet low in phytanic and pristanic acids were recommended to the patient at follow‐up. A gradual improvement was observed in liver dysfunction. The level of pristanic acid decreased with dietary therapy, but the patient's adherence to the diet also decreased after initial clinical improvement. At the age of 10, the Wechsler Intelligence Scale for Children‐Revised (WISC‐R) disclosed an intelligence quotient (IQ) of 87 (Verbal IQ, 88; Performance IQ, 89). She was 14 years old at the last out‐patient evaluation, and she had not adhered to the recommended restricted phytanic and pristanic acid diet. After 11 years of follow‐up, biochemical evaluation revealed elevated AST (67 U/L, nr: 5–40), ALT (51 U/L, nr: 10–40) levels, normal INR: 0.91 (nr: 0.8–1.25), elevated pristanic acid: 22.12 (nr: 0–1.5), and phytanic acid: 4.91 (nr: 0.42–3.77) levels.

**TABLE 1:** Clinical characteristics and laboratory features of our patients with AMACR deficiency.

Characteristic	Patient A1	Patient A2	Patient B1	Patient B2
Age	14 years	26 year	25 years	28 years
Sex	F	M	M	F
Parental consanguinity	First cousin	First cousin	First cousin	First cousin
Gestation week, birth weight	T, 3150 g	T, 3000 g	T, 3800 g	T, 3500 g
Age symptom onset/diagnosis	10 months/3 years	15/15 years	18/19 years	22/22 years
Initial symptoms	Coagulopathy, hepatic dysfunction	None, FS	Rhabdomyolysis	None, FS
Clinical presentation	Hepatic dysfunction	Hepatosteatosis	Rhabdomyolysis	Asymptomatic
Molecular findings	c.596G > A (hom)	c.596G > A (hom)	c.1006G > A (hom)	c.1006G > A (hom)
AST, U/L (nr: 5–40)	88	95	472	17
ALT, U/L (nr: 10–40)	70	90	248	15
CK, U/L (nr: 30–200)	230	278	**16 886**	77
Total cholesterol (mg/dL) (nr: 130–200)	96	107	109	116
Low‐density lipoprotein cholesterol (mg/dL) (nr: 100–130)	57	67	53	47
high‐density lipoprotein cholesterol (mg/dL) (nr > 40)	27	28	53	58
triglycerides (mg/dL) (nr:0–150)	57	74	49	67
Pristanic acid (μmol/L) (nr: 0–1.5)	**12.80**	**8.77**	**4.69**	**4.99**
Phytanic acid (μmol/L), (nr: 0.42–3.77)	1.98	2.29	1.31	1.21
C22:0 (μmol/L) (nr: 41.1–90.3)	31.75	55.54	24.50	50.13
C24:0 (μmol/L) (nr: 37.4–79.4)	49.59	42.75	21.40	39.71
C26:0 (μmol/L) (nr: 0.6–1.3)	0.82	0.54	0.56	0.49
C24:0/C22:0 (nr: 0.689–1.008)	1.69	0.77	0.87	0.79
C26:0/C22:0 (nr: 0.011–0.026)	0.03	0.01	0.02	0.01
Abdominal ultrasonography	Increased echogenicity of liver	Increased echogenicity of liver and hepatosteatosis	N	N
Liver biopsy	Hepatocellular degeneration and regeneration	Macrovesicular steatosis 20%	‐	‐
Cranial MRG	Nonspecific millimetric signal increase in right frontoparietal deep white matter	‐	N	‐
EMG	N	N	N	‐
Ocular findings	N	N	N	N

*Note*: Aberrant values are indicated in bold.

Abbreviations: EMG, electromyography; F, female; FS, family screening; hom, homozygous; M, male; MRG, magnetic resonance imaging; N, normal; nr, normal range; t, term.

### 2.2 Patient A2

The second patient who was the older brother of patient A1 was diagnosed at family screening at the age of 15 years. He had elevated liver transaminases and plasma pristanic acid levels. Physical examination was normal. There was no hepatosplenomegaly. Plasma AST (95 U/L, nr: 5–40), ALT (90 U/L, nr: 10–40), and pristanic acid 8.77 μmol/L (nr: 0–1.5) levels were elevated. Plasma cholesterol levels were low. Abdominal USG showed increased echogenicity of the liver and hepatosteatosis. Liver biopsy showed 20% macrovesicular steatosis. EMG was normal, and neuropsychological evaluation was unremarkable. Ophthalmologic examination was normal. Genetic analyses revealed homozygosity for variant c.596G>A (p.Cys20Tyr) on the *AMACR* gene. Dietary treatment with low phytanic and pristanic acids was recommended, but his dietary adherence was poor. At the time of the last evaluation at the age of 26 years, AST (77 U/L [nr: 5–40]), ALT (96 U/L [nr: 10–40]), and INR (0.91 [nr: 0.8–1.25]) were detected.

### 2.3 Patient B1

He was consulted for rhabdomyolysis at the age of 18 years. He was born at term, of consanguine Turkish parents. His psycho‐motor and physical development were normal with no hepatomegaly. He was a medical student. He had two acute attacks of rhabdomyolysis following strenuous exercise at the ages of 18 and 24 years during both of which plasma CK increased to 20 000 U/L. Arterial blood gas analyses, serum lactate, ammonia, urea, creatinine, total protein, albumin, metabolic screening including a dried blood spot analysis of amino acids and acylcarnitines, quantitative plasma amino acids, and urinary organic acids had revealed normal results. Plasma cholesterol levels were low. VLCFA analysis in plasma showed normal C26, C24, and C22 levels, and plasma pristanic acid was elevated (4.69 μmol/L, nr: 0–1.5). ECHO, EMG, abdominal USG, ophthalmological evaluation, and cranial MRI were normal. At asymptomatic periods, his CK levels were mildly elevated (316 U/L and 576 U/L, nr: 30–200). Measurement of bile acid intermediates in dried blood spot using ultra performance liquid chromatography‐MS/MS (UPLC‐MS/MS) showed striking elevation of unconjugated DHCA (1.25 μM, nr <0.02–0.05). Unconjugated THCA (0.08 μM, nr:0.0), glyco‐THCA (0.15 μM, nr <0.01), tauro‐THCA (1.17 μM, nr <0.07), and taurotetrahydroxycholestanoic acid (0.07 μM, n:0.0) were also higher than in controls. The conjugated C24 (normal) bile acids were within the control range. These findings were sufficient to confirm the diagnosis of a peroxisomal disorder affecting β‐oxidation of cholestenoic acids, probably AMACR deficiency. Multigene Next‐Generation Sequencing (NGS) panel was planned for the differential diagnosis of rhabdomyolysis. Homozygous c.1006G>A p.Ala336Thr, a novel variant in the *AMACR* gene was compatible with AMACR deficiency. The parents were carriers of this variant. A diet with low phytanic and pristanic acids was recommended, but the compliance was poor. He was 24 years old at the last out‐patient evaluation, and biochemical markers showed AST (16 U/L, nr: 5–40), ALT (24 U/L, nr: 10–40), CK (190 U/L, nr: 30–200), pristanic acid (3.74 μmol/L, nr: 0–1.5), and phytanic acid (0.92 μmol/L, nr: 0.42–3.77). After diagnosis, the patient had one more attack of rhabdomyolysis and did not develop any other system involvement.

### 2.4 Patient B2

She was the sister of patient B1 and was diagnosed at the age of 22 years during family screening. She had no complaints and no hospitalizations. Physical examination was normal. Laboratory workup revealed normal liver function tests—AST: 17 U/L (nr: 5–40), ALT: 15 U/L (nr: 10–40), and CK: 77 U/L (nr: 30–200). Plasma cholesterol levels were low. VLCFA in plasma showed normal C26, C24, and C22 levels, and plasma pristanic acid was elevated (4.99 μmol/L, nr: 0–1.5). ECHO, abdominal USG, and ophthalmological evaluations were normal. Measurement of bile acid intermediates in dried blood spot using UPLC‐MS/MS showed elevation of unconjugated DHCA (0.74 μM, nr < 0.02–0.05). Unconjugated THCA (0.01 μM, nr: 0.0), glyco‐THCA (0.06 μM, nr < 0.01), tauro‐THCA (0.24 μM, nr < 0.07), and taurotetrahydroxycholestanoic acid (0.13 μM, nr: 0.0) were also higher than in controls. The conjugated C24 (normal) bile acids were within the control range. Genetic analyses revealed a similar homozygosity for a novel variant c.1006G>A p.Ala336Thr on the *AMACR* gene as her brother. A diet with low phytanic and pristanic acid content was recommended, but the compliance was poor. She did not develop any new findings during the follow‐up for 6 years. At the time of the last admission at the age of 28 years, levels of AST (12 U/L, nr: 5–40), ALT (12 U/L, nr: 10–40), CK (49 U/L, nr: 30–200), pristanic acid (6.1 μmol/L, nr: 0–1.5), and phytanic acid (1.8 μmol/L, nr: 0.42–3.77) were detected. She had given birth by cesarean section at the age of 27 years without complications. Clinical characteristics and laboratory features of the patients are listed in Table 1. All study participants provided written informed consent.

## 3 DISCUSSION

We described four patients from two unrelated families; two of the new cases were diagnosed based on positive family history and had homozygous variants in the *AMACR* gene. The patients were followed for periods of 11 and 7 years within two sibling pairs at our center. Because of the rarity of AMACR deficiency, the understanding of its clinical spectrum is still evolving as infantile and late onset forms. All cases published with the infantile form had hepatic disease.^2^, ^4^, ^5^ Liver involvement in AMACR deficiency may include hepatomegaly, hepatosteatosis, and cholestasis. Patient A1 presented with hepatomegaly, hepatic dysfunction, and coagulopathy in infancy. Setchell et al. reported a patient with coagulopathy and mild cholestasis in the neonatal period.^2^ In patient A1, cholestasis was not observed. Gündüz et al. reported a case with the same variant as patients A1 and A2 presenting with mildly elevated liver enzymes as an isolated finding.^4^ This patient had no history of cholestasis, and the neurologic examination was normal. AMACR is an important diagnostic marker, which is highly expressed in several cancers.^6^ In the literature, a singular case of a 51‐year‐old patient diagnosed with AMACR deficiency was reported to have a liver tumor with a biopsy confirming a probable diagnosis of liver sarcoma.^7^ Patients diagnosed with AMACR deficiency should follow up for malignancies, especially hepatic carcinoma that may develop due to the metabolic disorder of the liver.

Patient B1 with the late‐onset form had a novel homozygous variant and presented with rhabdomyolysis without any other complaints. AMACR deficiency is an uncommon cause of rhabdomyolysis where only two cases have so far been reported.^8^, ^9^ Kapina et al. reported that neuroleptic malignant syndrome with recurrent episodes of rhabdomyolysis were clinical features of AMACR deficiency.^8^ In addition to rhabdomyolysis, this patient had developed seizures, febrile stroke‐like episode, hemiparesis, and hemi neglect syndrome later in life. Krett et al. reported a case study of a patient who received a diagnosis of bipolar disorder at the age of 16 and experienced subclinical seizures, as well as rhabdomyolysis attacks after the age of 40.^9^ After it was revealed that AMACR deficiency leads to rhabdomyolysis, the *AMACR* gene has been added to the rhabdomyolysis and metabolic myopathy panel. Furthermore, it is crucial to analyze VLCFA, phytanic acid, and pristanic acid levels in the differential diagnosis of rhabdomyolysis.

Another clinical manifestation of AMACR deficiency includes seizures, which may emerge at early adulthood and may be the initial symptom. On the other hand, previous publications showed that the patients may present with seizures in the fifth decade of life.^10^ Eleven patients (52%) had seizures in the literature. Nine of them had homozygosity for variant c.154T>C on the *AMACR* gene (Table 2).

**TABLE 2:** Clinical and molecular results of AMACR patients in the literature.

Study	Age at diagnosis/sex	Clinical findings	*AMACR*‐gene variant (homozygous)
Ferdinandusse et al.^1^	44 years/M 48 years/F 18 months/M	Adult‐onset sensory motor neuropathy, PR, developmental delay, seizures Demyelinating sensory motor polyneuropathy NA	c.154T>C c.154T>C c.320T>C
Van Veldhoven et al.^5^	1 years/F	Neonatal hepatitis, coagulopathy	‐
McLean et al.^19^	44 years/M	Learning difficulties, seizures, encephalopathy, optic atrophy, headache, PR	c.154T>C
Setchell et al.^2^	2.5 months/F	Cholestatic liver disease, coagulopathy	c.154T>C
Clarke et al.^18^	36 years/F	Cataract, migraine, depression, tremor, seizures, PR, encephalopathy	c.154T>C
Thompson et al.^20^	57 years/F	Seizures, neuropathy, relapsing encephalopathy, depression, hemiparesis, homonymous hemianopia	c.154T>C
Kapina et al.^8^	23 year/M	Schizophrenia, neuroleptic malignant syndrome, rhabdomyolysis, hemiparesis, hemineglect syndrome, retinopathy	c.559G>A
Smith et al.^10^	45 years/M	Seizures, relapsing encephalopathy, hemiparesis, PR	c.154T>C
Stewart et al.^21^	45 years/M	Seizures, encephalopathy, hemiparesis, altered mental status, PR	‐
Dick et al.^22^	58 years/M	Seizures, sensorimotor polyneuropathy, cerebellar ataxia	c.154T>C
Verhagen et al.^11^	9 months/M	Asymptomatic	5p13.3 deletion
Gündüz et al.^4^	10 months/M	Mild hepatosplenomegaly, elevated liver enzymes	c.596G>A
Alsalamah et al.^3^	16 years/M 19 years/F 22 years/F	Juvenile cholelithiasis, learning difficulties, subtle retinopathy Juvenile cholelithiasis, learning difficulties, subtle retinopathy Juvenile cholelithiasis, learning difficulties, subtle retinopathy	c.877T>C c.877T>C c.877T>C
Krett. et al.^9^	51 years/M	Rhabdomyolysis, depression, bipolar disorder, subclinical seizures	c.154T>C
Tanti et al.^23^	Seventh decade/F	Seizures, migraine, transient ischemic attack, encephalopathy, glaucoma and cataract, ataxia, tremor, central apnea	c.154T>C
Haugarvoll et al.^7^	30 years/M 33 years/F	Episodic elevation of serum creatine kinase, cataract, demyelinating peripheral neuropathy, type 2 diabetes mellitus, hepatosteatosis, PR, seizures, liver tumor Seizures, Type 2 diabetes mellitus, peripheral neuropathy, encephalopathy, cataract	c.367G>A c.367G>A
Patient A1	3 years/F	Hepatic dysfunction, coagulopathy, hypocholesterolemia	c.596G>A
Patient A2	15 years/M	Hepatosteatosis, hypocholesterolemia	c.596G>A
Patient B1	19 years/M	Rhabdomyolysis, hypocholesterolemia	c.1006G>A
Patient B2	22 years/F	Asymptomatic, hypocholesterolemia	c.1006G>A

Abbreviations: F, female; M, male; PR, pigmentary retinopathy.

Ocular features of AMACR deficiency include pigmentary retinopathy, cataract, and optic atrophy. Thirteen patients (62%) developed eye findings (Table 2). Eye findings of our patients were all normal. Alsalamah and Khan reported three siblings with AMACR deficiency who had no visual complaints.^3^ Although the appearance of the retina in ocular examination was normal, retinal multimodal imaging and electrophysiology revealed retinal dysfunction.

Clinical diagnosis of AMACR deficiency is challenging because of the wide spectrum of phenotypes and ages at presentation. In the literature, there are cases that were symptomatic in the fifth decades of life. Verhagen et al. reported a patient with oculocutaneous albinism type 4 and incidental finding of AMACR deficiency.^11^ The patient was asymptomatic but with elevated phytanic and pristanic acid, DHCA, and THCA, and homozygous 5p13.3 deletion encompassed *AMACR* genes. However, AMACR disease may also be asymptomatic, and as it is considered a very rare form of peroxisomal disorder, its prevalence may be underestimated.

All of our patients had low levels of plasma cholesterol, which is essential for normal brain development, myelination, and bile acid synthesis. Although hypocholesterolemia has not been mentioned in previously reported cases, it is known that reduced plasma cholesterol levels have been a consistent finding in patients with Zellweger spectrum. Deficiency of normal bile acids in the intestinal lumen leads to cholesterol malabsorption, but cholesterol synthesis may also be downregulated. While functional peroxisomes are essential for efficient cholesterol synthesis, its precise impact on specific pathways remains uncertain. Hypocholesterolemia may also manifest in other disorders, including abetalipoproteinemia types I and II, chylomicron retention disorder, PMM2‐congenital disorder of glycosylation, mevalonic aciduria, 3β‐hydroxy‐Δ5‐C27 steroid dehydrogenase deficiency, Smith–Lemli–Opitz syndrome, and Tangier disease.^12^, ^13^

A homozygous c.154T>C pathogenic variant of the *AMACR* gene is the most common variant (59%) in AMACR deficiency cases. Genotype–phenotype correlations have not yet been reported for AMACR deficiency, and the same variants may lead to different phenotypic manifestations. For example, the predominant variant c.154T>C has been described for both the early and late‐onset forms of the disorder.^10^ In AMACR deficiency, a phenotypic heterogeneity in the same family was not reported in the literature. Our two affected siblings manifested variable presentations. AMACR deficiency was described as both a mitochondrial and peroxisomal enzyme, so mitochondrial AMACR may also play a role in the phenotypic heterogeneity.

The c.1006G>A (p.Ala336Thr, rs746094231) variant was not related with disease before and was reported as very rare in populations (0.000004%, gnomAD).^14^ This missense variant caused an alanine‐to‐threonine (A to T) substitution at position 336 at the protein level. These amino acids are different in their chemical structure and properties, as well as their biological functions. The methyl side‐chain of alanine is nonreactive and is therefore hardly ever directly involved in protein function while threonine is a polar amino acid containing a hydroxyl group. For this variant, there are multiple lines of computational evidence to support a deleterious effect on the gene or gene product (conservation, evolutionary, etc.).^15^, ^16^ According to the American College of Medical Genetics and Genomics and the Association for Molecular Pathology variant classification, this variant is proposed as likely pathogenic, because it meets the criteria of population data (PM2), allelic data (PM3), segregation data (PP1), in‐silico predictions (PP3), and phenotype (PP4),^17^ (assessed by Franklin: https://franklin.genoox.com/).

Current treatment strategies for AMACR deficiency include diet therapy (low in pristanic and phytanic acids) and cholic acid supplementation. Patient A1 presented with hepatic dysfunction in infancy which improved with a diet low in phytanic and pristanic acid. Smith et al., also reported a patient who followed a low pristanic acid diet with observed clinical improvement but unchanged serum pristanic acid concentration.^10^ Clarke et al. reported a patient who had a low pristanic/phytanic acid diet for 2 years with no change in plasma pristanic acid or clinical condition.^18^ However, the current clinical experience is very limited due to the very few number of reported cases. Individualized treatment strategy for patients with AMACR deficiency needs to be further explored.

## 4 CONCLUSION

We aim to raise the awareness of AMACR deficiency by describing clinical, biochemical, and genetic features of the disease in different phenotypes. AMACR deficiency is usually described as an adult‐onset disorder, but better definition of clinical phenotypes and natural history may support earlier diagnosis and treatment. Early diagnosis is important as patients with this disorder may benefit from restricted dietary phytanic and pristanic acid intake. Follow up of liver involvement and protection from rhabdomyolysis are also important for this ultra‐rare disease.

## AUTHOR CONTRIBUTIONS

Arzu Selamioğlu conceptualized and designed the study and drafted the initial manuscript. Mehmet Cihan Balci designed the data collection instruments and critically reviewed and revised the manuscript. Meryem Karaca and Hacer Durmuş Tekçe followed the patient clinically and provided essential input into the manuscript. Asuman Gedikbaşı conducted the VLCFA analysis and genetic interpretation. Youssef Khalil, Rohit Hirachan and Peter Clayton analyzed bile acid intermediate metabolites. Peter Clayton revised the manuscript. Yeşim Gülşen Parman and Mübeccel Demirkol reviewed the manuscript. Gülden Gökçay critically supervised the whole study process. All authors read and approved the final manuscript. All authors have accepted responsibility for the entire content of this manuscript and approved its submission.

## CONFLICT OF INTEREST STATEMENT

The authors declare no conflict of interest.

## ETHICS STATEMENT

Ethics approval was not required because it is a retrospective case report. Istanbul University ethics committee made that decision.

## INFORMED CONSENT

All procedures followed were in accordance with the ethical standards of the responsible committee on human experimentation (institutional and national) and with the Helsinki Declaration of 1975, as revised in 2000.^5^ Informed consent was obtained from all patients for being included in the study.

## ANIMAL RIGHTS

All institutional and national guidelines for the care and use of laboratory animals were followed.

## Data Availability

All data generated or analyzed during this study are included in this study. Further enquiries can be directed to the corresponding author.
